# Converging toward a common speech code: imitative and perceptuo-motor recalibration processes in speech production

**DOI:** 10.3389/fpsyg.2013.00422

**Published:** 2013-07-11

**Authors:** Marc Sato, Krystyna Grabski, Maëva Garnier, Lionel Granjon, Jean-Luc Schwartz, Noël Nguyen

**Affiliations:** ^1^Grenoble Images Parole Signal Automatique-LAB, Département Parole and Cognition, Centre National de la Recherche Scientifique, Grenoble UniversitéGrenoble, France; ^2^Centre for Research on Brain, Language and Music, McGill UniversityMontreal, QC, Canada; ^3^Laboratoire Psychologie de la Perception, Centre National de la Recherche Scientifique, École Normale SupérieureParis, France; ^4^Laboratoire Parole and Langage, Centre National de la Recherche Scientifique, Aix-Marseille UniversitéAix-en-Provence, France

**Keywords:** phonetic convergence, imitation, speech production, speech perception, sensory-motor interactions, internal models, perceptual learning

## Abstract

Auditory and somatosensory systems play a key role in speech motor control. In the act of speaking, segmental speech movements are programmed to reach phonemic sensory goals, which in turn are used to estimate actual sensory feedback in order to further control production. The adult's tendency to automatically imitate a number of acoustic-phonetic characteristics in another speaker's speech however suggests that speech production not only relies on the intended phonemic sensory goals and actual sensory feedback but also on the processing of external speech inputs. These online adaptive changes in speech production, or phonetic convergence effects, are thought to facilitate conversational exchange by contributing to setting a common perceptuo-motor ground between the speaker and the listener. In line with previous studies on phonetic convergence, we here demonstrate, in a non-interactive situation of communication, online unintentional and voluntary imitative changes in relevant acoustic features of acoustic vowel targets (fundamental and first formant frequencies) during speech production and imitation. In addition, perceptuo-motor recalibration processes, or after-effects, occurred not only after vowel production and imitation but also after auditory categorization of the acoustic vowel targets. Altogether, these findings demonstrate adaptive plasticity of phonemic sensory-motor goals and suggest that, apart from sensory-motor knowledge, speech production continuously draws on perceptual learning from the external speech environment.

## Introduction

Speech production is a complex multistage motor process that requires phonetic encoding, initiation and coordination of sequences of supra-laryngeal and laryngeal movements produced by the combined actions of the pulmonary/respiratory system, the larynx and the vocal tract. Influential models of speech motor control postulate that auditory and somatosensory representations also play a key role in speech production. It is proposed that segmental speech movements are programmed to reach phonemic auditory and somatosensory goals, which in turn are used to estimate actual sensory inputs during speech production (for reviews, Perkell et al., 1997, 2000; Perrier, 2005, 2012; Guenther, 2006; Guenther and Vladusich, 2012; Perkell, 2012). The relationships between speech motor commands and sensory feedback are thought to be progressively learned by the central nervous system during native (and foreign) language acquisition, leading to the establishment of mature phonemic sensory-motor goals.

In adult/fluent speech production, a large number of studies employing manipulations of both somatosensory and auditory feedback also support the hypothesis that sensory feedback plays an important role in tuning speech motor control. For instance, transient transformations of both the auditory and somatosensory feedback, due to unexpected dynamical mechanical loading of supra-laryngeal articulators, result in on-line and rapid articulatory adjustments in speech production (Folkins and Abbs, 1975; Abbs and Gracco, 1984; Gracco and Abbs, 1985). Similarly, online modifications of the auditory feedback in its pitch (Elman, 1981; Burnett et al., 1998; Jones and Munhall, 2000), vowel formant frequencies (Houde and Jordan, 1998; Jones and Munhall, 2000; Houde et al., 2002; Purcell and Munhall, 2006a,b; Cai et al., 2011; Rochet-Capellan and Ostry, 2011, 2012) or fricative first spectral moment (Shiller et al., 2009, 2010) also induce compensatory changes in speech production. Finally, although auditory information is often assumed to be the dominant sensory modality, the integration of somatosensory information in the achievement of speech movements has also been demonstrated (Tremblay et al., 2003; Nasir and Ostry, 2006; Feng et al., 2011; Lametti et al., 2012). Importantly, these studies not only demonstrate online motor corrections to counteract the effect of perturbations, but also a persistence of those corrections (i.e., an after-effect) once the perceptual manipulation is removed (Houde and Jordan, 1998; Jones and Munhall, 2000; Houde et al., 2002; Tremblay et al., 2003; Nasir and Ostry, 2006; Purcell and Munhall, 2006b; Shiller et al., 2009). The fact that motor compensatory adjustments do not disappear immediately likely reflects a global temporary remapping, or re-calibration, of the sensory-motor relationships.

Due to the intrinsic temporal limitations of the biological feedback systems, the concepts of efference copy (von Holst and Mittelstaedt, 1950) and internal models (Francis and Wonham, 1976; Kawato et al., 1987) have been introduced in order to explain how the central nervous system rapidly reacts to perturbations and adjusts fine-grained motor parameters (Guenther, 1995; Perkell et al., 1997; Guenther et al., 1998; Houde and Jordan, 1998; for recent reviews, see Perkell et al., 2000; Guenther, 2006; Hickok et al., 2011; Houde and Nagarajan, 2011; Guenther and Vladusich, 2012; Hickok, 2012; Perkell, 2012; Perrier, 2012).

During language acquisition, perceptuo-motor goals that define successful speech motor acts are thought to be gradually explored and acquired in interaction with adult speakers (Kuhl and Meltzoff, 1996; Kuhl et al., 1997; Kuhl, 2004). The relationships between speech motor commands and sensory feedback signals are then progressively learned by the central nervous system, and stored in the form of an internal *forward* model. The internal forward model allows for the prediction of the sensory consequences of speech motor movements in relation with the intended sensory speech goals. These internal sensory predictions, generated prior to the actual motor execution and sensory feedback, can assist in speech motor control. In case of discrepancy between the internal sensory predictions and the actual sensory feedback, corrective motor commands are estimated in order to further control production. Such corrective motor commands from the internal forward model allow refining and updating the relationships between the intended sensory speech goals and the relevant sequence of motor commands, which are then stored in an internal *inverse* model. Once the inverse model has been learned, it is hypothesized that speech production, in mature/fluent speech and in normal circumstances, operates almost entirely under the internal inverse model and feedforward control mechanisms (for recent reviews, see Guenther and Vladusich, 2012; Perkell, 2012; Perrier, 2012). From that view, the intended phonemic sensory goal allows the internal inverse model to internally specify the relevant speech motor sequences, without involvement of the internal forward model and sensory feedback control mechanisms, thus compensating for the delay inherent in sensory feedback. On the other hand, sensory feedback can still be used for online corrective motor adjustments, in case of external perturbations, in the comparison between internal sensory predictions from the forward model and actual sensory inputs.

The above-mentioned studies and models demonstrate a key role of on-line auditory and somatosensory feedback control mechanisms in speech production and suggest that speech goals are defined in multi-dimensional motor, auditory and somatosensory spaces. However, for all their importance, these studies fail to reveal the extent to which speech perception and production systems may be truly integrated when speaking. First, individual differences in perceptual capacities may also act on speech production. From that view, a recent study on healthy adults, with no reported impairment of hearing or speech, demonstrates that individual differences in auditory discrimination abilities influence the degree to which speakers adapt to altered auditory feedback (Villacorta et al., 2007; but see Feng et al., 2011). Second, many studies of adaptation in speech production have focused primarily on the flexibility of motor processes, without regard for possible adaptive changes of phonemic sensory representations that are presumed to constitute the sensory goals of speech movements (except during language acquisition and the learning of internal models). However, two studies involving altered auditory or somatosensory feedback show compensatory changes not only in production of a speech sound, but also in its perception (Nasir and Ostry, 2009; Shiller et al., 2009). These results thus suggest plasticity of phonemic sensory representations in relation to adjustment of motor commands. Finally, the adult's tendency to automatically imitate a number of acoustic-phonetic characteristics in another speaker's speech suggests that speech production relies not only on the intended phonemic sensory goals and actual sensory feedback but also on the processing of external speech inputs.

In keeping with this later finding, the present study aimed at investigating adaptive plasticity of phonemic sensory-motor goals in speech production, based on either unintentional or voluntary vowel imitation. In addition to speech motor control, the working hypothesis of the present study capitalizes on previous studies on perceptual learning and speech imitation as well as on the theoretical proposal of a functional coupling between speech perception and action systems.

In this framework, it is worthwhile noting that speech and vocal imitation is one of the basic mechanisms governing the acquisition of spoken language by children (Kuhl and Meltzoff, 1996; Kuhl et al., 1997; Kuhl, 2004). In adults, unintentional speech imitation, or phonetic convergence, has been found to also occur in the course of a conversational interaction (for recent reviews, see Babel, 2009; Aubanel, 2011; Lelong, 2012). The behavior of each talker can evolve with respect to that of the other talker in two opposite directions: it may become more similar to the other talker's behavior (a phenomenon referred to as convergence) or more dissimilar. Convergence effects have been shown to be systematic and recurrent, and manifest themselves under many different forms, including posture (Shockley et al., 2003), head movements and facial expressions (Estow et al., 2007; Sato and Yoshikawa, 2007) and, regarding speech, vocal intensity (Natale, 1975; Gentilucci and Bernardis, 2007), speech rate (Giles et al., 1991; Bosshardt et al., 1997), voice onset time (Flege, 1987; Flege and Eefting, 1987; Sancier and Fowler, 1997; Fowler et al., 2008), fundamental frequency, and pitch curve (Gregory, 1986; Gregory et al., 1993; Bosshardt et al., 1997; Kappes et al., 2009; Babel and Bulatov, 2012), formant frequencies and spectral distributions (Gentilucci and Cattaneo, 2005; Delvaux and Soquet, 2007; Gentilucci and Bernardis, 2007; Aubanel and Nguyen, 2010; Lelong and Bailly, 2011). Apart from directly assessing phonetic convergence on acoustic parameters, other studies measured convergence by means of perceptual judgments, mostly using AXB tests (Goldinger, 1998; Goldinger and Azuma, 2004; Pardo, 2006; Pardo et al., 2010; Kim et al., 2011). Importantly, phonetic convergence has been shown to manifest in a variety of ways. Some involve natural settings, as during conversational exchange when exposure to the speech of others leads to phonetic convergence with that speech (Natale, 1975; Pardo, 2006; Aubanel and Nguyen, 2010; Pardo et al., 2010; Kim et al., 2011; Lelong and Bailly, 2011), or when exposure to a second language influences speech production of a native language, and vice-versa (Flege, 1987; Flege and Eefting, 1987; Sancier and Fowler, 1997; Fowler et al., 2008). Other involve non-interactive situations of communication, as when hearing and/or seeing a recorded speaker influences the production of similar or dissimilar speech sounds (Goldinger and Azuma, 2004; Gentilucci and Cattaneo, 2005; Delvaux and Soquet, 2007; Gentilucci and Bernardis, 2007; Kappes et al., 2009; Babel and Bulatov, 2012). Altogether, these phenomena of “speech accommodation” may facilitate conversational exchange by contributing to setting a common ground between speakers (Giles et al., 1991). In that respect, they may have the same effect as so-called alignment mechanisms, which are assumed to apply to linguistic representations at different levels between partners, in order for these partners to have a better joint understanding of what they are talking about (Garrod and Pickering, 2004; Pickering and Garrod, 2004, 2007).

Apart from social attunement, can phonetic convergence be also explained at a more basic sensory-motor level? In our view, phonetic convergence necessarily involves complex sensorimotor interactions that allow the speaker to compare or tune his/her own sensory and motor speech repertoire with the phonetic characteristics of the perceived utterance. Since phonetic convergence implies perception of speech sounds prior to actual speech production, phonetic convergence is likely to first rely on perceptual processing and learning from the external speech environment, leading to adaptive plasticity of phonemic sensory goals.

From that view, a significant body of speech perception research has demonstrated that sensory representations of speech sounds are flexible in response to changes in the sensory and linguistic aspects of speech input (e.g., Ladefoged and Broadbent, 1957; Miller and Liberman, 1979; Mann and Repp, 1980). In addition, studies on perceptual learning, or perceptual recalibration, have provided evidence for increased performance in speech perception/recognition and changes in perceptual representations after exposure to only a few speech sounds (e.g., Nygaard and Pisoni, 1998; Bertelson et al., 2003; Norris et al., 2003; Clarke and Garrett, 2004; Kraljic and Samuel, 2005, 2006, 2007; McQueen et al., 2006; Bradlow and Bent, 2008). In addition to perceptual learning, it is also to note that several psycholinguistic and neurobiological models of speech perception argue that phonetic interpretation of sensory speech inputs is determined, or at least partly constrained, by articulatory procedural knowledge (Liberman et al., 1967; Liberman and Mattingly, 1985; Fowler, 1986; Liberman and Whalen, 2000; Schwartz et al., 2002, 2012; Scott and Johnsrude, 2003; Callan et al., 2004; Galantucci et al., 2006; Wilson and Iacoboni, 2006; Skipper et al., 2007; Rauschecker and Scott, 2009). These models postulate that sensorimotor interactions play a key role in speech perception, with the motor system thought to partly constrain phonetic interpretation of the sensory inputs through the internal generation of candidate articulatory categories. Taken together, these studies and models thus suggest that listeners maintain perceptual and motor representations that incorporate fine-grained information about specific speech sounds, speakers, and situations. Hence, during speech production, phonetic convergence may arise from induced plasticity of phonemic sensory and motor representations, in relation to relevant adjustment of motor commands.

To extend the above-mentioned findings on phonetic convergence and to further test adaptive plasticity of phonemic sensory-motor goals in speech production, the present study aimed at investigating, in a non-interactive situation of communication, both unintentional and voluntary imitative changes in relevant acoustic features of acoustic vowel targets during speech production and imitation. A second goal of this study was to test offline perceptuo-motor recalibration processes (i.e., after-effects) after vowel production, imitation, and categorization.

## Methods

### Participants

Three groups of twenty-four healthy adults, native French speakers, participated in the production, imitation and categorization experiments (12 females and 12 males per group). In order to test possible relationships between phonetic convergence and voluntary imitation, a subgroup of 12 subjects (6 females and 6 males) participated in both the production and imitation experiments (see Procedure). All participants had normal or corrected-to-normal vision, and reported no history of speaking, hearing or motor disorders.

### Stimuli

Multiple utterances of /i/, /e/, and /ε/ steady-state French vowels were individually produced from a visual orthographic target and recorded by six native French speakers (3 females and 3 males) in a sound-attenuated room. In order to cover the typical range of *F*_0_ values during vowel production for male and female speakers, the six speakers were selected with respect to their largely distinct fundamental frequency (*F*_0_) values during vowel production (see below). None of the speakers participated in the three experiments.

Throughout this study, the focus was put on the main determinant of the voice characteristics that is *F*_0_, leaving aside a number of other possible acoustic parameters that could also provide targets for convergence phenomena (e.g., voice quality, *F*_0_ variations inside the spoken utterances, intensity, duration, etc.). In the same vein, the focus was comparatively put on one of the main characteristic of vowels' phonetic quality that is *F*_1_, considering that in the set of unrounded front vowels here used, *F*_1_ is both the basic cue to distinguishing these vowels from one another (see for example Ménard et al., 2002), and shows large variations from one French speaker to another (e.g., Ménard et al., 2008). This also leaves aside a number of other acoustic determinants of phonetic quality such as *F*_2_, but also *F*_3_ which is known to play an important role in the front unrounded region, particularly for /i/ and to a lesser extent for /e/. The choice to focus on acoustic variables a priori considered as the main characteristics in each domain seemed adequate in order to focus on major phenomena and escape from difficult—and largely unsolved—questions associated with the weighing of perceptual cues in a given perceptual domain.

One token of each vowel was selected per speaker and digitized in an individual sound file at a sampling rate of 44.1 kHz with 16-bit quantization recording. Using Praat software (Boersma and Weenink, 2013), each vowel was scaled to 75 dB and cut, at zero crossing points, from the vocalic onset to 250 ms following it. *F*_0_ and first formant (*F*_1_) values were then calculated for each vowel from a period defined as ±25 ms of the maximum peak intensity (see Table 1). With this procedure, the stimuli differed in *F*_0_ and *F*_1_ values according to both gender and speaker (mean *F*_0_ averaged across vowels: 100–120–136 Hz and 196–249–296 Hz for the three male and the three female speakers, respectively; mean *F*_1_ for /i/, /e/, and /ε/ vowels: 258–314–496 Hz and 285–414–646 Hz for the three male and the three female speakers, respectively).

**Table 1:** ***F*_0_ and *F*_1_ values of /i/, /e/, /ε/ target vowels according to the six recorded speakers (3 females/males)**.

**Vowel**	**Gender**	***F*_0_**			***F*_1_**		
		**S1**	**S2**	**S3**	**S1**	**S2**	**S3**
/i/	Female	210	251	288	285	269	301
/e/	Female	190	248	290	389	399	453
/ε/	Female	187	249	284	693	577	668
		**S4**	**S5**	**S6**	**S4**	**S5**	**S6**
/i/	Male	137	120	103	278	248	247
/e/	Male	139	120	98	390	324	228
ε/	Male	132	121	100	510	440	538

### Experimental procedure

**Figure 1:**
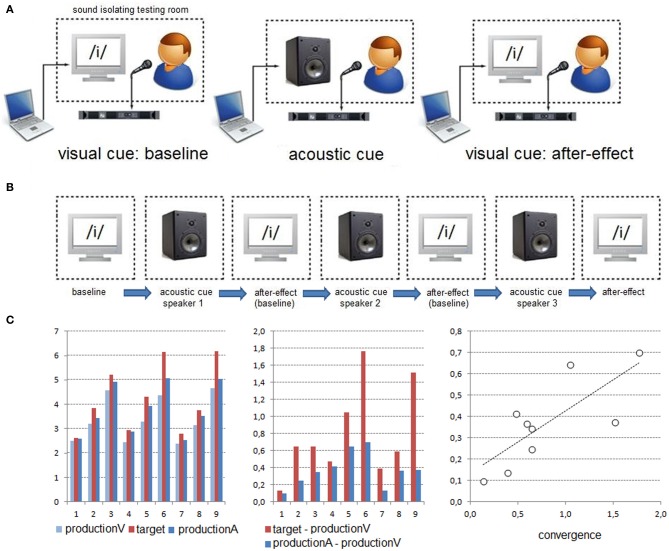
**Experimental procedure. (A)** In each block, participants were consecutively presented with orthographic, acoustic (previously recorded from a single speaker) and again orthographic vowel targets. The experimental design and apparatus were identical in the production, imitation and categorization experiments, except the task required during the presentation of the acoustic targets (i.e., vowel production, vowel imitation, and manual vowel categorization). This procedure allowed determining unintentional/automatic in the production experiment or voluntary imitative changes in the imitation experiment, as well as possible after-effects in the three experiments. **(B)** The experiments consisted of three blocks involving different acoustic targets (/i/, /e/, or /ε/ vowels from 3 males or females speakers). **(C)** Left: Example of production changes (here for *F*_1_) observed for one participant from the presentation of the visual target (productionV) to the presentation of the acoustic target (productionA). Middle-Right: A correlation was performed on 9 median data points (3 blocks × 3 vowels) in order to determine a possible relationship between acoustic changes of participants' productions (*Y*-axis) and the acoustic differences between the acoustic targets and the baseline (*X*-axis; see text for details).

The three experiments were carried out in a sound-proof room. Participants sat in front of a computer monitor at a distance of approximately 50 cm. The acoustic stimuli were presented at a comfortable sound level through a loudspeaker, with the same sound level set for all participants. The Presentation software (Neurobehavioral Systems, Albany, CA) was used to control the stimulus presentation during all experiments, and to record key responses in the categorization experiment (see below). All participants' productions were recorded for off-line analyses. The experimental design and apparatus were identical in all experiments, except the task required during the presentation of the acoustic stimuli (i.e., vowel production, vowel imitation and vowel categorization; see Figure 1).

- Production experiment: The experiment was designed to test phonetic convergence on acoustically presented vowels and to measure the magnitude of such online automatic imitative changes as well as possible offline perceptuo-motor recalibration due to phonetic convergence (after-effects). To this aim, participants were instructed to produce distinct vowels (/i/, /e/, or /ε/), one at a time, according to either a visual orthographic or an acoustic vowel target. Importantly, no instructions to “repeat” or to “imitate” the acoustic targets were given to the participants. Moreover, all participants were naive as to the purpose of the experiment. A block design was used where participants produced vowels according first to orthographic targets (baseline), then to acoustic targets (phonetic convergence) and finally to orthographic targets (after-effect). This procedure allowed comparing participants' productions 1) between the first presentation of the orthographic targets and the following presentation of the acoustic targets in order to determine possible convergence effects on *F*_0_ and *F*_1_ values according to the acoustic targets and 2) between the first and last presentations of the orthographic targets in order to determine possible after-effects.
- Imitation experiment: To compare phonetic convergence and voluntary imitation of the acoustic vowels, the second group of participants performed the same experiment except that they were explicitly asked to imitate the acoustic targets. The only indication given to the participants was to imitate the voice characteristics of the perceived speaker.
- Categorization experiment: The third experiment was designed to test whether after-effects can occur without prior unintentional/automatic or voluntary vowel imitation but after auditory categorization of the acoustic targets. To this aim, participants were instructed to produce vowels according to the orthographic targets and to manually categorize the acoustic vowel targets, without overt production. During the categorization task, participants were instructed to produce a motor response by pressing with their right hand, one of three keys corresponding to the /i/, /e/, or /ε/ vowels, respectively.

Each experiment consisted of three experimental blocks, involving the acoustic targets previously recorded by either the three female or the three male speakers. In each block, the /i/, /e/, and /ε/ acoustic targets were related to a single speaker. With this procedure, *F*_0_ values of the vowel targets remained similar within each block while *F*_1_ varied according to each vowel type. The block order (across the three speakers) was fully counterbalanced across participants. In each experiment, six female and six male participants were presented with acoustic targets from the female speakers and six female and six male participants were presented with acoustic targets from the male speakers. This procedure allowed testing possible differences in imitative changes and after-effects depending on participant's and speaker's acoustic space congruency (i.e., female/female and male/male vs. female/male and male/female participants/speakers).

Each experimental block consisted of the orthographic presentation of the three vowels (presented 5 times in a random order) then the acoustic presentation of the three vowels (randomly presented 10 times) and finally the orthographic presentation of the three vowels (randomly presented 5 times). Since perceptual learning from the external speech environment likely operated throughout the experiment, the last orthographic presentation of the vowels served as the first sub-block in the following experimental block. In each sub-block, each trial started with an orthographic or an acoustic target for 250 ms, a blank screen for 500 ms, a fixation cue (the “+” symbol) presented in the middle of the screen for 250 ms, and ended with a blank screen for 2000 ms. In order to limit possible close-shadowing effects (Porter and Lubker, 1980), participants were instructed to produce their response only after the presentation of the “+” symbol. Hence, the intertrial interval was 3 s.

The total duration of each experiment was around 10 min. The experiments were preceded by a brief training session. A debriefing was carried out at the end of each experiment. Importantly, none of the participants reported having voluntarily imitated the acoustic stimuli in the production experiment. Note that the subgroup of subjects who participated in both the production and imitation experiments, always first performed the production experiment first.

### Acoustic analyses

All acoustic analyses were performed using Praat software. A semi-automatic procedure was first devised for segmenting participants' recorded vowels (8640 utterances). For each participant, the procedure involved the automatic segmentation of each vowel based on an intensity and duration algorithm detection. Based on minimal duration and low intensity energy parameters, the algorithm automatically identified pauses between each vowel and set the vowel's boundaries on that basis. If necessary, these boundaries were hand-corrected, based on waveform and spectrogram information. Omissions, wrong productions and hesitations were manually identified and removed from the analyses. Finally, for each vowel, *F*_0_ and *F*_1_ values were calculated from a period defined as ±25 ms of the maximum peak intensity of the sound file.

The mean percentage of errors was 2.8, 1.2, and 1.2% in the production, imitation, and categorization experiments, respectively, with no participant exceeding the error limit of 10%. For each experiment, median *F*_0_ and *F*_1_ values calculated on all participants' productions confirmed a standard distribution for the /i/, /e/, and /ε/ French vowels, with differences mainly due to gender (see Table 2).

**Table 2:** **Median *F*_0_ and *F*_1_ values of /i/, /e/, /ε/ produced vowels averaged over all participants' productions according to gender in Experiments A–C**.

**Vowel**	**Gender**	**Experiment A**		**Experiment B**		**Experiment C**	
		***F*_0_**	***F*_1_**	***F*_0_**	***F*_1_**	***F*_0_**	***F*_1_**
/i/	Female	225	277	221	295	222	276
/e/	Female	220	416	216	417	215	407
/ε/	Female	214	613	211	610	210	607
/i/	Male	128	277	124	270	130	269
/e/	Male	125	372	120	366	126	365
/ε/	Male	123	508	119	522	125	545

## Results

For each participant and each sub-block, median *F*_0_ and *F*_1_ values were first computed for the /i/, /e/, and /ε/ vowels and expressed in bark [i.e., arctan(0.00076*f*) + 3.5 arctan((*f*/7500)^2^); Zwicker and Fastl, 1990]. For each experiment, median *F*_0_ and *F*_1_ exceeding ±2 standard deviations (*SD*; computed on the set of median values for the 24 participants) were removed from the analyses.

### Phonetic convergence and voluntary imitation (production and imitation experiments, see Figure 2)

**Figure 2:**
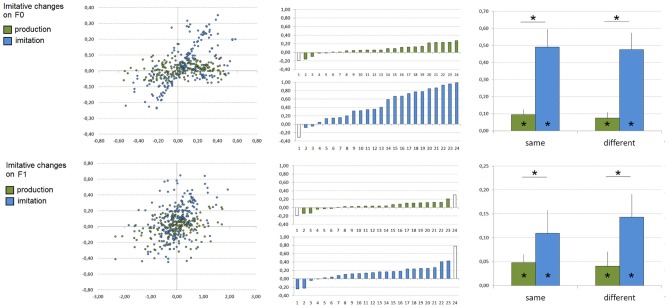
**Imitative changes observed during vowel production (in green) and imitation (in blue) on *F*_0_ (top) and *F*_1_ (bottom) values**. Left: All correlation-points based on 6480 utterances related to participants' imitative changes during the presentation of acoustic targets compared to their baseline productions (*X*-axis: acoustic target minus subject's production during the presentation of visual targets, *Y*-axis: subjects' production during the presentation of acoustic targets minus subject's production during the presentation of visual targets; all values are expressed in bark). Middle: Individual slope coefficients related to participants' imitative changes according to the task (*x*-axes are subjects, ordered by increasing slope coefficient; slope coefficients exceeding ±2 SD are represented in white and were removed from the analyses). Right: Mean slope coefficients related to participants' imitative changes according to the task and the acoustic space congruency (error bars represent standard error of the mean). ^*^Significant effects (*p* < 0.05) are indicated.

We here tested whether unintentional and voluntary imitation would result in shifting *F*_0_ and/or *F*_1_ toward the corresponding value for the acoustic target. To this aim, we first calculated acoustic changes of participants' productions between the presentation of acoustic targets and visual targets (baseline). For each participant and block, median *F*_0_ and *F*_1_ values produced in the baseline (i.e., median *F*_0_ and *F*_1_ values produced in the preceding sub-block during the presentation of the corresponding orthographic targets) were subtracted from those produced during the presentation of each type of acoustic targets (i.e., /i/, /e/, or /ε/). Next, we calculated acoustic changes between the acoustic targets and the baseline. These two sets of data, calculated on both *F*_0_ and *F*_1_ values, were then correlated in order to determine a possible relationship between acoustic changes of participants' productions and the acoustic differences between the acoustic targets and the baseline (see Figure 1C). For each participant, one set of 9 correlation-points (from 3 blocks and 3 vowels) was therefore calculated for both *F*_0_ and *F*_1_ and one single subject slope coefficient for each acoustic parameter was estimated from these values by means of linear regressions. In order to keep the data sets homogeneous, slope coefficients exceeding ±2 SD were removed from the following analyses (corresponding to one participant in both the production and imitation experiments for *F*_0_, and two and one participants in the production and imitation experiments, respectively, for *F*_1_, see Figure 2).

For both *F*_0_ and *F*_1_ slope coefficients, the remaining data were entered into analyses of variance (ANOVA) with the experiment (phonetic convergence, imitation) and the acoustic space congruency (same vs. different gender of the model speaker and the participant) as between-subject variables. In addition, individual one-tailed *t*-tests were performed for each experiment in order to test whether the mean slope coefficient was significantly superior to zero. Finally, in order to test whether imitative changes on *F*_0_ and *F*_1_ may correlate, a Pearson's correlation analysis was performed between single subject slope coefficients on *F*_0_ and *F*_1_ for each experiment.

For *F*_0_, ANOVA on single subject slope coefficients showed a significant effect of the task [*F*_(1, 42)_ = 27.16, *p* < 0.001], with stronger imitative changes according to the acoustic targets during the imitation task compared to the production task (mean slope coefficients of 0.08 and 0.48 in the production and imitation experiments). No effect of the acoustic space congruency [*F*_(1, 42)_ = 0.05] nor task × acoustic-space congruency interaction [*F*_(1, 42)_ = 0.01] were however observed. In addition, slope coefficients differed significantly from zero in both the production [*t*_(22)_ = 3.99, *p* < 0.001] and imitation [*t*_(22)_ = 7.11, *p* < 0.001] experiments.

For *F*_1_, there was also a significant effect of the task [*F*_(1, 41)_ = 4.95, *p* < 0.04], with stronger imitative changes during the imitation task compared to the production task (mean slope coefficients of 0.04 and 0.13 in the production and imitation experiments). As for *F*_0_, no effect of the acoustic space congruency [*F*_(1, 41)_ = 0.24] nor task × acoustic-space congruency interaction [*F*_(1, 41)_ = 0.45] were observed. Slope coefficients also differed significantly from zero in both the production [*t*_(21)_ = 2.78, *p* < 0.02] and imitation [*t*_(22)_ = 4.21, *p* < 0.001] experiments.

In addition, Pearson's correlation analyses showed no significant correlation between single subject slope coefficients observed for imitative changes on *F*_0_ and *F*_1_ in both the production (*r* = 0.08, slope = 0.06) and imitation (*r* = 0.03, slope = 0.01) experiments.

In sum, for both *F*_0_ and *F*_1_ values, these results demonstrate online imitative changes according to the acoustic vowel targets during production and imitation tasks, with stronger imitative changes in the voluntary vowel imitation task and a lower, albeit significant, phonetic convergence effect in the vowel production task. Interestingly, these effects were observed independently of the participant and speaker acoustic space congruency. Finally, it is worthwhile noting the large variability across participants, especially in the production task.

### After-effects (production, imitation and categorization experiments, see Figure 3)

**Figure 3:**
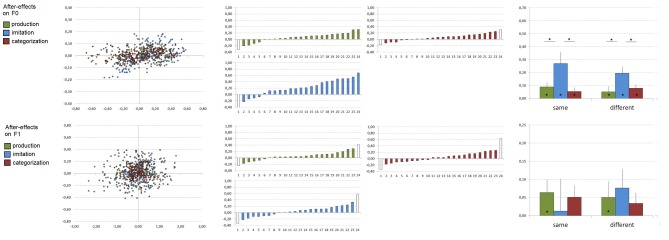
**After-effects observed after vowel production (in green), imitation (in blue) and manual categorization (in red) on *F*_0_ (top) and *F*_1_ (bottom) values**. Left: All correlation-points based on 4320 utterances related to participants' imitative changes during the presentation of acoustic targets compared to their baseline productions (*X*-axis: acoustic target minus subject's production during the first presentation of visual targets, *Y*-axis: subjects' production during the second presentation of visual targets minus subject's production during the first presentation of visual targets; all values are expressed in bark. Middle: Individual slope coefficients related to participants' after-effects according to the task (*x*-axes are subjects, ordered by increasing slope coefficient; slope coefficients exceeding ±2 SD are represented in white and were removed from the analyses). Right: Mean slope coefficients related to participants' after-effects according to the task and the acoustic space congruency (error bars represent standard error of the mean). ^*^Significant effects (*p* < 0.05) are indicated.

We also tested possible perceptuo-motor recalibration, i.e., after-effects, compared to the participant's baseline. For each participant, block and vowel, median *F*_0_ and *F*_1_ values produced during the preceding baseline were subtracted from those produced during the second presentation of the orthographic targets. As previously, for each participant, one set of 9 correlation-points (from 3 blocks and 3 vowels) were therefore calculated for both *F*_0_ and *F*_1_ (see Figure 3) and single subject slope coefficients were estimated from these values by means of linear regressions. Slope coefficients exceeding ±2 SD were removed from the following analyses (corresponding to one, two and two participants in the production, imitation, and categorization experiments, respectively, for *F*_0_, and two participants in the production, imitation, and categorization experiments for *F*_1_, see Figure 3).

For both *F*_0_ and *F*_1_ slope coefficients, the remaining data were entered into ANOVA with the experiment (phonetic convergence, imitation, and auditory categorization) and the acoustic space congruency (same, different) as between-subject variables. In addition, individual one-tailed *t*-tests were performed for each experiment in order to test whether the mean slope coefficient was significantly superior to zero. As previously, in order to test whether after-effects on *F*_0_ and *F*_1_ may correlate, a Pearson's correlation analysis was performed between single subject slope coefficients on *F*_0_ and *F*_1_ for each experiment.

For *F*_0_, ANOVA on single subject slope coefficients showed a significant effect of the task [*F*_(1, 42)_ = 6.98, *p* < 0.005], with stronger after-effects related to the acoustic targets after the imitation task compared to the production and categorization tasks (mean slope coefficients of 0.07, 0.23, and 0.07 in the production, imitation, and categorization experiments). No effect of the acoustic space congruency [*F*_(1, 42)_ = 0.50] nor task × acoustic-space congruency interaction [*F*_(1, 42)_ = 0.49] were however observed. In addition, slope coefficients differed significantly from zero in both the production [*t*_(22)_ = 2.85, *p* < 0.01], imitation [*t*_(22)_ = 4.92, *p* < 0.001] and categorization [*t*_(21)_ = 3.44, *p* < 0.005] experiments.

For F_1_, no significant effect of the task [*F*_(1, 41)_ = 0.08], of the acoustic space congruency [*F*_(1, 41)_ = 0.11] nor interaction [*F*_(1, 41)_ = 0.63] were observed. Slope coefficients differed significantly from zero in the production experiment [mean slope coefficient of 0.06; *t*_(21)_ = 2.59, *p* < 0.02] but not in the imitation [mean slope coefficient of 0.04; *t*_(21)_ = 1.83, *p* = 0.07] and categorization [mean slope coefficient of 0.04; *t*_(21)_ = 1.88, *p* = 0.07] experiments.

In addition, Pearson's correlation analyses showed no significant correlation between single subject slope coefficients observed for after-effects on *F*_0_ and *F*_1_ in both the production (*r* = −0.08, slope = −0.07), imitation (*r* = 0.10, slope = 0.06) and categorization (*r* = 0.11, slope = 0.18) experiments.

Hence, for *F*_0_, offline perceptuo-motor recalibration processes were observed after vowel production, imitation, and categorization of the acoustic targets, with a stronger after-effect after voluntary vowel imitation and lower, albeit significant, after-effects after vowel production and categorization. Furthermore, these effects were observed independently of the participant and speaker acoustic space congruency. For *F*_1_, a small after-effect was only observed after vowel production, although there was also a trend in the same direction after vowel imitation and categorization. Finally, as for online adaptive changes, there was a large variability across participants in all tasks.

### Relationships between imitative changes and after-effects (production and imitation experiments, see Figure 4)

**Figure 4:**
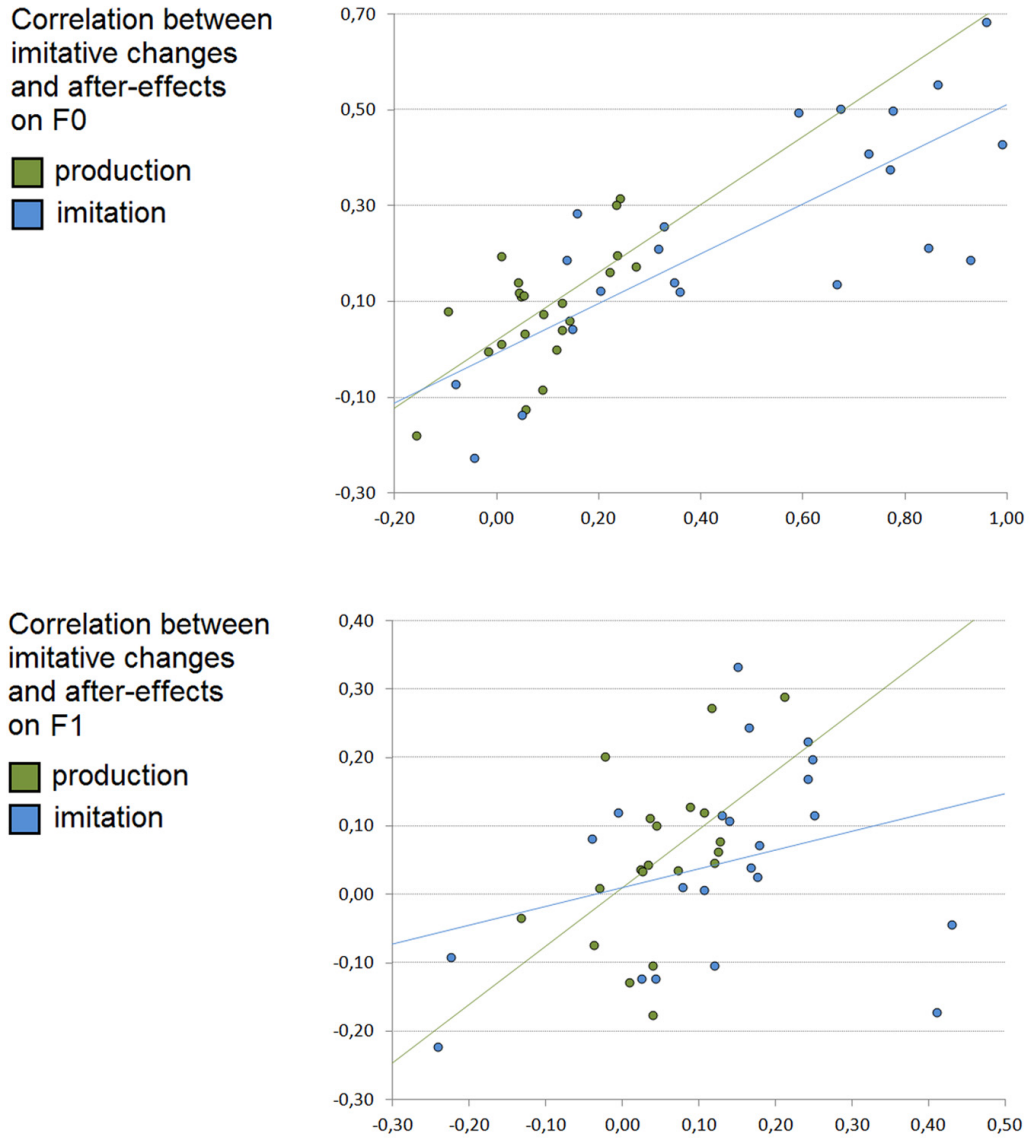
**Correlation between imitative changes and after-effects after vowel production (in green) and imitation (in blue) on *F*_0_ (top) and *F*_1_ (bottom) values**. *X*-axis: Individual slope coefficients related to online participants' imitative changes, *Y*-axis: individual slope coefficients related to participants' after-effects.

In order to test whether imitative changes and after-effects in the production and imitation experiments may correlate, Pearson's correlation analyses were performed for both *F*_0_ and *F*_1_ between single subject slope coefficients corresponding to the imitative changes and to the after-effects (see Figure 4). As previously, slope coefficients exceeding ±2 SD were removed from the analyses (corresponding to two participants in both experiments for *F*_0_, and four and two participants in the production and imitation experiments for *F*_1_, see Figure 4).

For *F*_0_, the Pearson's correlation analysis showed a significant correlation between single subject slope coefficients observed for imitative changes and for after-effects in both the production (*r* = 0.64, slope = 0.71, *p* < 0.005) and imitation (*r* = 0.78, slope = 0.52, *p* < 0.001) experiments.

For *F*_1_, the Pearson's correlation analysis also showed a significant correlation between single subject slope coefficients observed for imitative changes and for after-effects in the production experiment (*r* = 0.53, slope = 0.85, *p* < 0.03) but not in the imitation experiment (*r* = 0.31, slope = 0.27).

### Relationships between phonetic convergence and voluntary imitation (production and imitation experiments)

In order to test whether phonetic convergence and voluntary imitation in the production and imitation experiments may correlate for the subgroup of subjects who participated in both experiments, Pearson's correlation analyses were performed for both *F*_0_ and *F*_1_ between single subject slope coefficients corresponding to the convergence and imitative changes in the two experiments (see Figure 4). As previously, slope coefficients exceeding ±2 SD were removed from the analyses (corresponding to two participants for *F*_0_, and one for *F*_1_, see Figure 4).

The Pearson's correlation analysis showed no significant correlation between single subject slope coefficients observed for imitative changes in the two experiments for *F*_0_ (*r* = −0.24, slope = −0.55) and *F*_1_ (*r* = 0.19, slope = 0.38).

## Discussion

Influential models of speech motor control postulate a key role for on-line auditory and somatosensory feedback control mechanisms in speech production and highlight the sensory-motor nature of speech representations. However, studies on phonetic convergence suggest that speech production relies not only on phonemic sensory goals and actual sensory feedback but also on the processing of external speech inputs. In line with these findings, the present study demonstrates, in a non-interactive situation of communication, both unintentional and voluntary imitative changes in fundamental and first formant frequencies of acoustic vowel targets during speech production and imitation tasks. Offline perceptuo-motor recalibration processes on fundamental frequency—and possibly, marginally, for first formant frequency—were also observed after vowel production, imitation, and categorization of the acoustic targets. In addition, while a significant correlation was observed between imitative changes and after-effects in both vowel production and imitation tasks, no correlation occurred between phonetic convergence effects and voluntary imitative changes for the subgroup of subjects who participated in both experiments on vowel production and imitation. Altogether, these results demonstrate adaptive plasticity of phonemic sensory-motor goals and suggest that speech production draws on both sensory-motor knowledge and perceptual learning of the external speech environment.

### Online unintentional and voluntary imitative changes

Phonetic convergence effects have been initially explored during natural and interactive settings, notably during conversational exchanges between two speaking partners (Pardo, 2006; Aubanel and Nguyen, 2010; Pardo et al., 2010; Kim et al., 2011; Lelong and Bailly, 2011). This led to the hypothesis that “speech accommodation” may facilitate conversational exchange by contributing to setting a common ground between speakers (Giles et al., 1991; see also Garrod and Pickering, 2004; Pickering and Garrod, 2004, 2007). However, other studies conducted in a non-interactive laboratory setting, as when hearing and/or seeing a recorded speaker influences the production of similar or dissimilar speech sounds (Goldinger and Azuma, 2004; Gentilucci and Cattaneo, 2005; Delvaux and Soquet, 2007; Gentilucci and Bernardis, 2007; Kappes et al., 2009; Babel and Bulatov, 2012), indicate that convergence mechanisms do not depend on mutual adjustments and social attunement only. In our view, these later studies provide powerful evidence that, unless hindered by higher-order socio-psychological factors, phonetic convergence is a highly automatized process (for a review, see Delvaux and Soquet, 2007) that may also be triggered by low-level sensory and motor adaptive processes.

Based on *F*_0_ and *F*_1_ acoustic analyses of a large corpus of recorded vowels, the present study replicates and extends phonetic convergence effects previously observed on fundamental frequency (Gregory, 1986; Gregory et al., 1993; Bosshardt et al., 1997; Kappes et al., 2009; Babel and Bulatov, 2012) and on formant frequencies and spectral distributions (Gentilucci and Cattaneo, 2005; Delvaux and Soquet, 2007; Gentilucci and Bernardis, 2007; Aubanel and Nguyen, 2010; Lelong and Bailly, 2011). First, online imitative changes on both *F*_0_ and *F*_1_ in relation to the acoustic vowel targets were observed in a non-interactive situation of communication during the production task, with none of the participants reporting having voluntarily imitated the acoustic stimuli. Second, although previous studies usually involved the production of words or sentences (Goldinger, 1998; Goldinger and Azuma, 2004; Pardo, 2006; Delvaux and Soquet, 2007; Kappes et al., 2009; Aubanel and Nguyen, 2010; Pardo et al., 2010; Kim et al., 2011; Lelong and Bailly, 2011; Babel and Bulatov, 2012), adaptive changes were here observed during vowel production thus minimizing lexical/semantic processing (for phonetic convergence effects on *F*_0_ and/or *F*_1_ during syllable or non-word production, see Gentilucci and Cattaneo, 2005; Gentilucci and Bernardis, 2007; Kappes et al., 2009). Altogether, these findings thus suggest that phonetic convergence may also derive from unintentional and automatic adaptive sensory-motor speech mechanisms. However, it is worthwhile noting that, although significant at the group level, the magnitude of these adaptive changes was rather small (mean slope coefficients of 0.08 and of 0.04 for *F*_0_ and *F*_1_, respectively) and quite variable across participants (individual slope coefficients ranging from −0.16 to 0.27 and from −0.14 to 0.21 for *F*_0_ and *F*_1_, respectively). In addition, although phonetic convergence was attested for both *F*_0_ and *F*_1_, adaptive changes were twice lower for *F*_1_. In the experiments, however, *F*_0_ values of the vowel targets remained similar within each block while *F*_1_ varied according to each vowel type. Although sensory-motor and convergence mechanisms are likely to differ for these acoustic parameters at the acoustical, biomechanical and neurobiological levels, it appears difficult to speculate on these observed differences. Finally, although gender effects have been previously observed on phonetic convergence (Pardo, 2006; Pardo et al., 2010; Babel and Bulatov, 2012), the exact nature of this mediation remains unclear and may depend on both specific experimental designs and/or “macro” social mechanisms, out of the scope of this study. Given the limited number of participants in each sub-experimental group condition (i.e., six female and six male participants presented with acoustic targets from the female speakers, and six female and six male participants presented with acoustic targets from the male speakers), we rather focused on the participant and speaker acoustic space congruency. Phonetic convergence was observed independently of the participant and speaker acoustic space congruency, a result suggesting that phonetic convergence on vowels and in a non-interactive situation of communication is pervasive and not strongly influenced by the acoustic distance between the participant and the model speaker.

To compare phonetic convergence and voluntary imitation of the acoustic vowels, a second group of participants performed the same experiment except that they were explicitly asked to imitate the acoustic targets. As expected, stronger online imitative changes according to the acoustic vowel targets were observed during voluntary imitation (mean slope coefficients of 0.48 vs. 0.08 for *F*_0_ and 0.13 vs. 0.04 for *F*_1_, for the imitation and production tasks, respectively). As in the production tasks, imitative changes were however quite variable across participants (individual slope coefficients ranging from −0.8 to 0.99 and from −0.24 to 0.43 for *F*_0_ and *F*_1_, respectively). In addition, no significant correlation between phonetic convergence and voluntary imitation on both *F*_0_ and *F*_1_ were observed for the subgroup of subjects who participated in both the production and imitation task. Interestingly, although not significant, the slope coefficient for *F*_0_ appears nevertheless quite high (mean slope coefficients of −0.55). Hence, although this last result does not indicate any significant correlation, possible dependencies between phonetic convergence and voluntary imitation have to be further investigated in future studies.

### Perceptuo-motor recalibration processes

Interestingly, previous studies showed clear evidence of post-exposure imitation, with experimental designs and long-lasting effects that preclude strategic explanations (Goldinger and Azuma, 2004; Pardo, 2006; Delvaux and Soquet, 2007). In these studies, phonetic convergence was first attested during the production of auditorily presented words in a non-interactive situation of communication. Offline adaptation to the acoustic targets was however observed in post-tests occurring either immediately (Pardo, 2006; Delvaux and Soquet, 2007) or even conducted one week after the production task (Goldinger and Azuma, 2004; see also Goldinger, 1998 using a close-shadowing task). These latter findings suggest that long-term memory to some extent preserves detailed traces of the auditorily presented words and thus support episodic/exemplar theories of word processing assuming that paralinguistic details of a spoken word are stored together as a memory trace (e.g., Nygaard et al., 1994; Goldinger, 1996, 1998; Nygaard and Pisoni, 1998), although hybrid models combining abstract phonological representations with episodic memory traces are also consistent with these results (e.g., McQueen et al., 2006; Pierrehumbert, 2006). Importantly, Pardo (2006) and Delvaux and Soquet (2007) also propose that these observed phonetic convergence and associated long-term adaptive changes may be at the source of gradual diachronic changes of a phonological system in a community.

In line with these studies, offline perceptuo-motor recalibration processes were here observed for *F*_0_ after vowel production, imitation and auditory categorization of the acoustic targets, with a stronger after-effect observed after voluntary vowel imitation. The fact that after-effects equally occurred following prior vowel production and perceptual categorization of the acoustic targets likely suggests that these effects rely on perceptual processing and learning from the acoustic targets, without the need for a specific motor learning stage. As for online imitative changes, these effects were observed independently of the participant and speaker acoustic space congruency and, although significant at the group level, the magnitude of these after-effects was rather small (mean slope coefficients of 0.07, 0.23, and 0.07 for the production, imitation and categorization tasks, respectively) and quite variable across participants (individual slope coefficients ranging from −0.20 to 0.32, from −0.23 to 0.68 and from −0.12 to 0.25 for the production, imitation and categorization tasks, respectively). As expected, a significant correlation between single subject slope coefficients for imitative changes and after-effects was also observed in both the production and imitation tasks.

For *F*_1_, a small after-effect was only observed after vowel production (although there was a trend after vowel imitation and categorization), with a significant correlation between single subject slope coefficients for imitative changes and after-effects. The after-effect for *F*_1_ in the production task and the trend found in the imitation and categorization tasks were therefore observed despite *F*_1_ values of the acoustic targets varying according to each vowel type in each block. Finally, it is also interesting to note that for *F*_1_ the imitation task did not provide stronger after-effects as compared to the other tasks. More intriguing is the very low after-effect observed in the imitation tasks when subjects and targets were of the same gender, a phenomenon for which we do have no clear explanation yet.

### Perceptuo-motor learning and internal models of speech production

Altogether, our results demonstrate adaptive plasticity of phonemic sensory-motor goals in a non-interactive situation of communication, without lexical/semantic processing of the acoustic targets. Although they appear in line with previous studies on phonetic convergence and do not contradict the theoretical proposal that adaptive changes in speech production facilitate conversational exchanges between speaking partners, these results demonstrate that, in addition to social attunement and lexical/semantic processing, convergence effects may also be triggered by low-level sensory and motor adaptive speech processes. From that point of view, future studies on phonetic convergence contrasting interactive and non-interactive laboratory settings will be of great interest to further determine whether social interactions might enhance imitative changes.

Together with previous studies on phonetic convergence and imitation, the observed adaptive plasticity of phonemic sensory-motor goals sheds an important light on speech motor control and internal models of speech production (for reviews, Perkell et al., 1997, 2000; Perrier, 2005, 2012; Guenther, 2006; Guenther and Vladusich, 2012; Perkell, 2012). As previously noted, these models postulate that auditory and somatosensory systems play a key role in speech motor control and that speech goals are defined in multi-dimensional motor, auditory, and somatosensory spaces. However, they mainly focus on the flexibility of motor processes, without regard for possible adaptive changes of phonemic sensory representations that are presumed to constitute the sensory goals of speech movements. Convergence and perceptuo-motor recalibration processes however demonstrate that speech production relies not only on the intended phonemic sensory goals and actual sensory feedback but also on the processing of external speech inputs. In our view, these effects are based on complex sensorimotor interactions, allowing the speaker to compare or tune his/her own sensory and motor speech repertoire with the phonetic characteristics of the perceived utterance, and leading to perceptuo-motor learning from the external speech environment. During speech production, phonetic convergence and after-effects may therefore arise from induced plasticity of phonemic sensory and motor representations, in relation to relevant adjustment of motor commands. Convergence effects are thus of considerable interest since they suggest that speech motor goals are continuously updated in response to changes in the sensory and linguistic aspects of speech inputs. Hence, as also advocated by Perkell (2012), adaptive processes, likely to modify online, to a certain extent, sensory speech representations, will have to be taken into account in future versions of speech motor control models.

From that view, there is now considerable neurobiological evidence that sensorimotor interactions play a key role in both speech perception and speech production. In line with internal models of speech production, modulation of neural responses observed within the auditory and somatosensory cortices when speaking are thought to reflect feedback control mechanisms in which predicted sensory consequences of the speech-motor act are compared with actual sensory input in order to further control production (Guenther, 2006; Tian and Poeppel, 2010; Hickok et al., 2011; Houde and Nagarajan, 2011; Price et al., 2011; Guenther and Vladusich, 2012; Hickok, 2012). In addition, it has been suggested that motor activity during speech perception partly constrains phonetic interpretation of the sensory inputs through the internal generation of candidate articulatory categories (Callan et al., 2004; Wilson and Iacoboni, 2006; Skipper et al., 2007; Poeppel et al., 2008; Rauschecker and Scott, 2009; Hickok et al., 2011; Rauschecker, 2011). From these models, perceptuo-motor learning and plasticity of phonemic goals induced by convergence and sensory-motor adaptive processes might depend on both a ventral and dorsal stream (Guenther, 2006; Hickok and Poeppel, 2007; Rauschecker and Scott, 2009; Hickok et al., 2011; Rauschecker, 2011; Guenther and Vladusich, 2012; Hickok, 2012; see also Grabski et al., 2013 for recent brain-imaging evidence that vowel production and perception both rely on these dorsal and ventral streams). The ventral stream (“what”) is supposed to be in charge for phonological and lexical processing, and thought to be localized in the anterior part of the superior temporal gyrus/sulcus (Scott and Johnsrude, 2003; Rauschecker and Scott, 2009; Rauschecker, 2011) or in the posterior part of the middle temporal gyrus and superior temporal sulcus (Hickok and Poeppel, 2007). The dorsal stream (“how”) would deal with sensory-motor mapping between sensory speech representations in the auditory temporal and somatosensory parietal cortices and articulatory representations in the ventral premotor cortex and the posterior part of the inferior frontal gyrus, with sensorimotor interaction converging in the supramarginal gyrus (Rauschecker and Scott, 2009; Rauschecker, 2011) or in area SPT (a brain region within the planum temporale near the parieto-temporal junction; Hickok and Poeppel, 2007). In line with the involvement of both the dorsal and ventral streams in imitative changes in speech production, recent studies using repetition, shadowing or voluntary imitation tasks have provided evidence for a neuro-functional/neuro-anatomical signature of speech imitation ability, mostly relying on the superior temporal gyrus, the premotor cortex and the inferior parietal lobule (Peschke et al., 2009; Irwin et al., 2011; Reiterer et al., 2011; Mashal et al., 2012). From these findings, the neural basis of low-level sensory and motor adaptive speech processes involved in phonetic convergence and perceptuo-motor recalibration processes remains to be investigated in future studies.

### Conflict of interest statement

The authors declare that the research was conducted in the absence of any commercial or financial relationships that could be construed as a potential conflict of interest.

## References

[B1] Abbs J. H. Gracco V. L. (1984). Control of complex motor gestures: orofacial muscle responses to load perturbations of lip during speech. J. Neurophysiol. 51, 705–723 6716120 10.1152/jn.1984.51.4.705

[B2] Aubanel V. (2011). Variation Phonologique Régionale en Interaction Conversationnelle. Doctoral Dissertation, Aix-Marseille University.

[B3] Aubanel V. Nguyen N. (2010). Automatic recognition of regional phonological variation in conversational interaction. Speech Commun. 52, 577–586 10.1016/j.specom.2010.02.008

[B4] Babel M. (2009). Phonetic and Social Selectivity in Speech Accommodation. Doctoral Dissertation, University of California, Berkeley.

[B5] Babel M. Bulatov D. (2012). The role of fundamental frequency in phonetic accommodation. Lang. Speech 55, 231–248 10.1177/0023830911417695 22783633

[B6] Bertelson P. Vroomen J. De Gelder B. (2003). Visual recalibration of auditory speech identification: a mcgurk aftereffect. Psychol. Sci. 14, 592–597 10.1046/j.0956-7976.2003.psci_1470.x 14629691

[B7] Boersma P. Weenink D. (2013). Praat: Doing Phonetics by Computer [Computer program]. Version 5.3.42. Available online at: http://www.praat.org/ (Accessed 2 March, 2013).

[B8] Bosshardt H. G. Sappok C. Knipschild M. Hölscher C. (1997). Spontaneous imitation of fundamental frequency and speech rate by nonstutterers and stutterers. J. Psycholinguist. Res. 26, 425–448 10.1023/A:1025030120016 9232010

[B9] Bradlow A. R. Bent T. (2008). Perceptual adaptation to non-native speech. Cognition 106, 707–729 10.1016/j.cognition.2007.04.005 17532315 PMC2213510

[B10] Burnett T. A. Freedland M. B. Larson C. R. Hain T. C. (1998). Voice F0 responses to manipulations in pitch feedback. J. Acoust. Soc. Am. 103, 3153–3161 10.1121/1.423073 9637026

[B11] Cai S. Ghosh S. S. Guenther F. H. Perkell J. S. (2011). Focal manipulations of formant trajectories reveal a role of auditory feedback in the online control of both within-syllable and between-syllable speech timing. J. Neurosci. 31, 16483–16490 10.1523/JNEUROSCI.3653-11.2011 22072698 PMC3268045

[B12] Callan D. E. Jones J. A. Callan A. M. Akahane-Yamada R. (2004). Phonetic perceptual identification by native- and second-language speakers differentially activates brain regions involved with acoustic phonetic processing and those involved with articulatory-auditory/orosensory internal models. Neuroimage 22, 1182–1194 10.1016/j.neuroimage.2004.03.006 15219590

[B13] Clarke C. M. Garrett M. F. (2004). Rapid adaptation to foreign accented English. J. Acoust. Soc. Am. 116, 3647–3658 10.1121/1.1815131 15658715

[B14] Delvaux V. Soquet A. (2007). The influence of ambient speech on adult speech productions through unintentional imitation. Phonetica 64, 145–173 10.1159/000107914 17914281

[B15] Elman J. L. (1981). Effects of frequency- shifted feedback on the pitch of vocal productions. J. Acoust. Soc. Am. 70, 45–50 10.1121/1.386580 7264071

[B16] Estow S. Jamieson J. P. Yates J. R. (2007). Self-monitoring and mimicry of positive and negative social behaviors. J. Res. Pers. 41, 425–433 10.1016/j.jrp.2006.05.003

[B17] Feng Y. Gracco V. L. Max L. (2011). Integration of auditory and somatosensory error signals in the neural control of speech movements. J. Neurophysiol. 106, 667–679 10.1152/jn.00638.2010 21562187 PMC3154803

[B18] Flege J. E. (1987). The production of “new” and “similar” phones in a foreign language: Evidence for the effect of equivalence classification. J. Phon. 15, 47–65

[B19] Flege J. E. Eefting W. (1987). Cross-language switching in stop consonant perception and production by Dutch speakers of English. Speech Commun. 6, 185–202 10.1016/0167-6393(87)90025-2

[B20] Folkins J. W. Abbs J. H. (1975). Lip and jaw motor control during speech: responses to resistive loading of the jaw. J. Speech Hear. Res. 18, 207–219 1127904 10.1044/jshr.1801.207

[B21] Fowler C. (1986). An event approach to the study of speech perception from a direct-realist perspective. J. Phon. 14, 3–28

[B22] Fowler C. A. Sramko V. Ostry D. J. Rowland S. A. Halle P. (2008). Cross language phonetic influences on the speech of French-English bilinguals. J. Phon. 36, 649–663 10.1016/j.wocn.2008.04.001 19802325 PMC2598425

[B23] Francis B. A. Wonham W. M. (1976). The internal model principle of control theory. Automatica 12, 457–651 10.1016/0005-1098(76)90006-6

[B24] Galantucci B. Fowler C. A. Turvey M. T. (2006). The motor theory of speech perception reviewed. Psychon. Bull. Rev. 13, 361–377 10.3758/BF03193857 17048719 PMC2746041

[B25] Garrod S. Pickering M. J. (2004). Why is conversation so easy? Trends Cogn. Sci. 8, 8–11 10.1016/j.tics.2003.10.016 14697397

[B26] Gentilucci M. Bernardis P. (2007). Imitation during phoneme production. Neuropsychologia 45, 608–615 10.1016/j.neuropsychologia.2006.04.004 16698051

[B27] Gentilucci M. Cattaneo L. (2005). Automatic audiovisual integration in speech perception. Exp. Brain Res. 167, 66–75 10.1007/s00221-005-0008-z 16034571

[B28] Giles H. Coupland N. Coupland J. (1991). Accommodation theory: communication, context, and consequence, in Contexts of Accommodation: Developments in Applied Sociolinguistics, eds Giles H. Coupland N. Coupland J. (Cambridge, UK: Cambridge University Press), 1–68 10.1017/CBO9780511663673.001

[B29] Goldinger S. D. (1996). Words and voices: episodic traces in spoken word identification and recognition memory. J. Exp. Psychol. Learn. Mem. Cogn. 22, 1166–1183 10.1037/0278-7393.22.5.1166 8926483

[B30] Goldinger S. D. (1998). Echoes of echoes? An episodic theory of lexical access. Psychol. Rev. 105, 251–279 10.1037/0033-295X.105.2.251 9577239

[B31] Goldinger S. D. Azuma T. (2004). Episodic memory reflected in printed word naming. Psychon. Bull. Rev. 11, 716–722 10.3758/BF03196625 15581123

[B32] Grabski K. Schwartz J. L. Lamalle L. Vilain C. Vallée N. Baciu M. (2013). Shared and distinct neural correlates of vowel perception and production. J. Neurolinguist. 26, 384–408 10.1016/j.jneuroling.2012.11.00316956772

[B33] Gracco V. L. Abbs J. H. (1985). Dynamic control of the perioral system during speech: kinematic analyses of autogenic and nonautogenic sensorimotor processes. J. Neurophysiol. 54, 418–432 4031995 10.1152/jn.1985.54.2.418

[B34] Gregory S. W. (1986). Social psychological implications of voice frequency correlations: analyzing conversation partner adaptation by computer. Soc. Psychol. Q. 49, 237–246 10.2307/2786806

[B35] Gregory S. W. Webster S. Huang G. (1993). Voice pitch and amplitude convergence as a metric of quality in dyadic interviews. Lang. Commun. 13, 195–217 10.1016/0271-5309(93)90026-J

[B36] Guenther F. H. (1995). Speech sound acquisition, coarticulation, and rate effects in a neural network model of speech production. Psychol. Rev. 102, 594–621 10.1037/0033-295X.102.3.594 7624456

[B37] Guenther F. H. (2006). Cortical interactions underlying the production of speech sounds. J. Commun. Disord. 39, 350–365 10.1016/j.jcomdis.2006.06.013 16887139

[B38] Guenther F. H. Hampson M. Johnson D. (1998). A theoretical investigation of reference frames for the planning of speech movements. Psychol. Rev. 105, 611–633 10.1037/0033-295X.105.4.611-633 9830375

[B39] Guenther F. H. Vladusich T. (2012). A neural theory of speech acquisition and production. J. Neurolinguist. 25, 408–422 10.1016/j.jneuroling.2009.08.006 22711978 PMC3375605

[B40] Hickok G. (2012). Computational neuroanatomy of speech production. Nat. Rev. Neurosci. 13, 135–145 22218206 10.1038/nrn3158 PMC5367153

[B41] Hickok G. Houde J. Rong F. (2011). Sensorimotor integration in speech processing: computational basis and neural organization. Neuron 69, 407–422 10.1016/j.neuron.2011.01.019 21315253 PMC3057382

[B42] Hickok G. Poeppel D. (2007). The cortical organization of speech processing. Nat. Rev. Neurosci. 8, 393–402 10.1038/nrn2113 17431404

[B43] Houde J. F. Jordan M. I. (1998). Sensorimotor adaptation in speech production. Science 279, 1213–1216 10.1126/science.279.5354.1213 9469813

[B44] Houde J. F. Nagarajan S. S. (2011). Speech production as state feedback control. Front. Hum. Neurosci. 5:82 10.3389/fnhum.2011.00082 22046152 PMC3200525

[B45] Houde J. F. Nagarajan S. S. Sekihara K. Merzenich M. M. (2002). Modulation of the auditory cortex during speech: an MEG study. J. Cogn. Neurosci. 14, 1125–1138 10.1162/089892902760807140 12495520

[B46] Irwin J. R. Frost S. J. Menci W. E. Chen H. Fowler C. A. (2011). Functional activation for imitation of seen and heard speech. J. Neurolinguist. 24, 611–618 10.1016/j.jneuroling.2011.05.001 21966094 PMC3182484

[B47] Jones J. A. Munhall K. G. (2000). Perceptual calibration of F0 production: evidence from feedback perturbation. J. Acoust. Soc. Am. 108, 1246–1251 10.1121/1.1288414 11008824

[B48] Kappes J. Baumgaertner A. Peschke C. Ziegler W. (2009). Unintended imitation in nonword repetition. Brain Lang. 111, 140–151 10.1016/j.bandl.2009.08.008 19811813

[B49] Kawato M. Furukawa K. Suzuki R. (1987). A hierarchical neural network model for the control and learning of voluntary movements. Biol. Cybern. 56, 1–17 3676355 10.1007/BF00364149

[B50] Kim M. Horton W. S. Bradlow A. R. (2011). Phonetic convergence in spontaneous conversations as a function of interlocutor language distance. Lab. Phonol. 2, 125–156 10.1515/labphon.2011.004 23637712 PMC3638967

[B51] Kraljic T. Samuel A. G. (2005). Perceptual learning for speech: is there a return to normal? Cogn. Psychol. 51, 141–178 10.1016/j.cogpsych.2005.05.001 16095588

[B52] Kraljic T. Samuel A. G. (2006). Generalization in perceptual learning for speech. Psychon. Bull. Rev. 13, 262–268 10.3758/BF03193841 16892992

[B53] Kraljic T. Samuel A. G. (2007). Perceptual adjustments to multiple speakers. J. Mem. Lang. 56, 1–15 10.1016/j.jml.2006.07.010

[B54] Kuhl P. K. (2004). Early language acquisition: cracking the speech code. Nat. Rev. Neurosci. 5, 831–843 10.1038/nrn1533 15496861

[B55] Kuhl P. K. Andruski J. E. Chistovich I. A. Chistovich L. A. Kozhevnikova E. V. Ryskina V. L. (1997). Cross-language analysis of phonetic units in language addressed to infants. Science 277, 684–686 10.1126/science.277.5326.684 9235890

[B56] Kuhl P. K. Meltzoff A. N. (1996). Infant vocalizations in response to speech: vocal imitation and developmental change. J. Acoust. Soc. Am. 100, 2425–2438 10.1121/1.417951 8865648 PMC3651031

[B57] Ladefoged P. Broadbent D. E. (1957). Information conveyed by vowels. J. Acoust. Soc. Am. 29, 98–104 10.1121/1.1908694 2525139

[B58] Lametti D. R. Nasir S. Ostry D. J. (2012). Sensory preference in speech production revealed by simultaneous alteration of auditory and somatosensory feedback. J. Neurosci. 32, 9351–9359 10.1523/JNEUROSCI.0404-12.2012 22764242 PMC3404292

[B59] Lelong A. (2012). Convergence Phonétique en Interaction. Doctoral Dissertation, Grenoble University.

[B60] Lelong A. Bailly G. (2011). Study of the phenomenon of phonetic convergence thanks to speech dominoes, in Analysis of Verbal and Nonverbal Communication and Enactment: the Processing Issue, eds Esposito A. Vinciarelli A. Vicsi K. Pelachaud C. Nijholt A. (Grenoble: Springer Verlag), 280–293

[B61] Liberman A. M. Cooper F. S. Shankweiler D. P. Studdert-Kennedy M. (1967). Perception of the speech code. Psychol. Rev. 74, 431–461 10.1037/h0020279 4170865

[B62] Liberman A. M. Mattingly I. G. (1985). The motor theory of speech perception revised. Cognition 21, 1–36 10.1016/0010-0277(85)90021-6 4075760

[B63] Liberman A. M. Whalen D. H. (2000). On the relation of speech to language. Trends Cogn. Sci. 3, 254–264 10.1016/s1364-6613(00)01471-6 10782105

[B64] Mann V. A. Repp B. H. (1980). Influence of vocalic context on perception of the [zh]-[s] distinction. Percept. Psychophys. 28, 213–228 10.3758/BF03204377 7432999

[B65] Mashal N. Solodkin A. Dick A. S. Chen E. E. Smal S. L. (2012). A network model of observation and imitation of speech? Front. Psychol. 3:84 10.3389/fpsyg.2012.00084 22470360 PMC3312271

[B66] McQueen J. M. Norris D. Cutler A. (2006). The dynamic nature of speech perception. Lang. Speech 49, 101–112 10.1177/00238309060490010601 16922064

[B67] Ménard L. Schwartz J. L. Aubin J. (2008). Invariance and variability in the production of the height feature in french vowels. Speech Commun. 50, 14–28 10.1016/j.specom.2007.06.004

[B68] Ménard L. Schwartz J. L. Boë L. J. Kandel S. Vallée N. (2002). Auditory normalization: of French vowels synthesized by an articulatory model simulating growth from birth to adulthood. J. Acoust. Soc. Am. 111, 1892–1905 10.1121/1.1459467 12002872

[B69] Miller J. L. Liberman A. M. (1979). Some effects of later-occurring information on the perception of stop consonant and semivowel. Percept. Psychophys. 25, 457–465 10.3758/BF03213823 492910

[B70] Nasir S. M. Ostry D. J. (2006). Somatosensory precision in speech production. Curr. Biol. 16, 1918–1923 10.1016/j.cub.2006.07.069 17027488

[B70a] Nasir S. M. Ostry D. J. (2009). Auditory plasticity and speech motor learning. Proc. Natl. Acad. Sci. U.S.A. 106, 20470–20475 10.1073/pnas.0907032106 19884506 PMC2771744

[B71] Natale M. (1975). Convergence of mean vocal intensity in dyadic communication as a function of social desirability. J. Pers. Soc. Psychol. 32, 790–804 10.1037/0022-3514.32.5.790

[B72] Norris D. McQueen J. M. Cutler A. (2003). Perceptual learning in speech. Cogn. Psychol. 47, 204–238 10.1016/S0010-0285(03)00006-9 12948518

[B73] Nygaard L. C. Pisoni D. B. (1998). Talker-specific learning in speech perception. Percept. Psychophys. 60, 355–376 10.3758/BF03206860 9599989

[B74] Nygaard L. C. Sommers M. S. Pisoni D. B. (1994). Speech perception as a talker contingent process. Psychol. Sci. 5, 42–45 10.1111/j.1467-9280.1994.tb00612.x 21526138 PMC3081685

[B75] Pardo J. S. (2006). On phonetic convergence during conversational interaction. J. Acoust. Soc. Am. 119, 2382–2393 10.1121/1.2178720 16642851

[B76] Pardo J. S. Jay I. C. Krauss R. M. (2010). Conversationnal role influences speech imitation. Atten. Percept. Psychophys. 72, 2254–2264 21097867 10.3758/bf03196699

[B77] Perkell J. S. (2012). Movement goals and feedback and feedforward control mechanisms in speech production. J. Neurolinguist. 25, 382–407 10.1016/j.jneuroling.2010.02.011 22661828 PMC3361736

[B78] Perkell J. S. Guenther F. H. Lane H. Matthies L. M. Perrier P. Vick J. (2000). A theory of speech motor control and supporting data from speakers with normal hearing and with profound hearing loss. J. Phon. 28, 233–272 10.1006/jpho.2000.0116

[B79] Perkell J. S. Matthies M. L. Lane H. Guenther F. H. Wilhelms-Tricarico R. Wozniak J. (1997). Speech motor control: acoustic goals, saturation effects, auditory feedback and internal models. Speech Commun. 22, 227–250 10.1016/S0167-6393(97)00026-5

[B80] Perrier P. (2005). Control and representations in speech production. ZAS Papers Linguist. 40, 109–132

[B81] Perrier P. (2012). Gesture planning integrating knowledge of the motor plant's dynamics: a literature review from motor control and speech motor control, in Speech Planning and Dynamics, eds Fuchs S. Weirich M. Pape D. Perrier P. (Frankfurt: Peter Lang), 191–238

[B82] Peschke C. Ziegler W. Kappes J. Baumgaertner A. (2009). Auditory-motor integration during fast repetition: the neuronal correlates of shadowing. Neuroimage 47, 392–402 10.1016/j.neuroimage.2009.03.061 19345269

[B83] Pickering M. J. Garrod S. (2004). Towards a mechanistic psychology of dialogue. Behav. Brain Sci. 27, 169–190 10.1017/S0140525X04000056 15595235

[B84] Pickering M. J. Garrod S. (2007). Do people use language production to make predictions during comprehension? Trends Cogn. Sci. 11, 105–110 10.1016/j.tics.2006.12.002 17254833

[B85] Pierrehumbert J. (2006). The next toolkit. J. Phon. 34, 516–530 10.1016/j.wocn.2006.06.003

[B86] Poeppel D. Idsardi W. J. van Wassenhove V. (2008). Speech perception at the interface of neurobiology and linguistics. Philos. Trans. R. Soc. Lond. B Biol. Sci. 363, 1071–1086 10.1098/rstb.2007.2160 17890189 PMC2606797

[B87] Porter R. J. Lubker J. F. (1980). Rapid reproduction of vowel sequences: evidence for a fast and direct acoustic motoric linkage in speech. J. Speech Hear. Res. 23, 593–602 7421161

[B88] Price C. J. Crinion J. T. MacSweeney M. (2011). A generative model of speech production in Broca's and Wernicke's areas. Front. Psychol. 2:237 10.3389/fpsyg.2011.00237 21954392 PMC3174393

[B89] Purcell D. W. Munhall K. G. (2006a) Compensation following real-time manipulation of formants in isolated vowels. J. Acoust. Soc. Am. 119, 2288–2297 10.1121/1.2173514 16642842

[B90] Purcell D. W. Munhall K. G. (2006b) Adaptive control of vowel formant frequency: evidence from real-time formant manipulation. J. Acoust. Soc. Am. 120, 966–977 10.1121/1.2217714 16938984

[B91] Rauschecker J. P. (2011). An expanded role for the dorsal auditory pathway in sensorimotor control and integration. Hear. Res. 271, 16–25 10.1016/j.heares.2010.09.001 20850511 PMC3021714

[B92] Rauschecker J. P. Scott S. K. (2009). Maps and streams in the auditory cortex: Nonhuman primates illuminate human speech processing. Nat. Neurosci. 12, 718–724 10.1038/nn.2331 19471271 PMC2846110

[B93] Reiterer S. M. Hu X. Erb M. Rota G. Nardo D. Grodd W. (2011). Individual differences in audio-vocal speech imitation aptitude in late bilinguals: functional neuro-imaging and brain morphology. Front. Psychol. 2:271. 10.3389/fpsyg.2011.00271 22059077 PMC3203549

[B94] Rochet-Capellan A. Ostry D. J. (2011). Simultaneous acquisition of multiple auditorymotor transformations in speech. J. Neurosci. 31, 2657–2662 10.1523/JNEUROSCI.6020-10.2011 21325534 PMC3079285

[B95] Rochet-Capellan A. Ostry D. J. (2012). Nonhomogeneous transfer reveals specificity in speech motor learning. J. Neurophysiol. 107, 1711–1717 10.1152/jn.00773.2011 22190628 PMC3311670

[B96a] Sancier M. L. Fowler C. A. (1997). Gestural drift in a bilingual speaker of Brazilian Portuguese and English. J. Phon. 25, 421–436 10.1006/jpho.1997.0051

[B96] Sato W. Yoshikawa S. (2007). Spontaneous facial mimicry in response to dynamic facial expressions. Cognition 104, 1–18 10.1016/j.cognition.2006.05.001 16780824

[B97] Schwartz J. L. Abry C. Boë L. J. Cathiard M. A. (2002). Phonology in a theory of perception-for-action-control, in Phonology: from Phonetics to Cognition, eds Durand J. Lacks B. (Oxford: Oxford University Press,), 240–280

[B98] Schwartz J. L. Ménard L. Basirat A. Sato M. (2012). The Perception for Action Control Theory (PACT): a perceptuo-motor theory of speech perception. J. Neurolinguist. 25, 336–354 10.1016/j.jneuroling.2009.12.004

[B99] Scott S. K. Johnsrude I. S. (2003). The neuroanatomical and functional organization of speech perception. Trends Neurosci. 26, 100–107 10.1016/S0166-2236(02)00037-1 12536133

[B100] Shiller D. M. Gracco V. L. Rvachew S. (2010). Auditory-motor learning during speech production in 9-11 year-old children. PLoS ONE 5:e12975 10.1371/journal.pone.0012975 20886033 PMC2945760

[B101] Shiller D. M. Sato M. Gracco V. L. Baum S. (2009). Perceptual recalibration of speech sounds following speech motor learning. J. Acoust. Soc. Am. 125, 1103–1113 10.1121/1.3058638 19206885

[B102] Shockley K. Santana M. V. Fowler C. A. (2003). Mutual interpersonal postural constraints are involved in cooperative conversation. J. Exp. Psychol. Hum. Percpt. Perform. 29, 326–332 12760618 10.1037/0096-1523.29.2.326

[B103] Skipper J. I. Van Wassenhove V. Nusbaum H. C. Small S. L. (2007). Hearing lips and seeing voices: how cortical areas supporting speech production mediate audiovisual speech perception. Cereb. Cortex 17, 2387–2399 10.1093/cercor/bhl147 17218482 PMC2896890

[B104] Tian X. Poeppel D. (2010). Mental imagery of speech and movement implicates the dynamics of internal forward models. Front. Psychol. 1:166 10.3389/fpsyg.2010.00166 21897822 PMC3158430

[B105] Tremblay S. Shiller D. M. Ostry D. J. (2003). Somatosensory basis of speech production. Nature 423, 866–869 10.1038/nature01710 12815431

[B106] Villacorta V. M. Perkell J. S. Guenther F. H. (2007). Sensorimotor adaptation to feedback perturbations of vowels acoustics and its relation to perception. J. Acoust. Soc. Am. 122, 2306–2319 10.1121/1.2773966 17902866

[B107] von Holst E. Mittelstaedt H. (1950). Das Reafferenzprinzip. Wechselwirkungen zwischen Zentralnervensystem und Peripherie. Naturwissenchaften 37, 464–476 10.1007/BF00622503

[B108] Wilson S. M. Iacoboni M. (2006). Neural responses to non-native phonemes varying in producibility: evidence for the sensorimotor nature of speech perception. Neuroimage 33, 316–325 10.1016/j.neuroimage.2006.05.032 16919478

[B109] Zwicker E. Fastl H. (1990). Psychoacoustics – Facts and Models. Heidelberg: Springer-Verlag

